# Small temperature variations are a key regulator of reproductive growth and assimilate storage in oil palm (*Elaeis guineensis*)

**DOI:** 10.1038/s41598-019-57170-8

**Published:** 2020-01-20

**Authors:** Naoki Tani, Zubaidah Aimi Abdul Hamid, Natra Joseph, Othman Sulaiman, Rokiah Hashim, Takamitsu Arai, Akiko Satake, Toshiaki Kondo, Akihiko Kosugi

**Affiliations:** 1 Forestry Division, Japan International Research Center for Agricultural Sciences (JIRCAS), Ohwashi, Tsukuba, Ibaraki 305-8686 Japan; 2 Faculty of Life and Environmental Sciences, University of Tsukuba, Tennodai, Tsukuba, Ibaraki 305-8577 Japan; 3 Bioresource, Paper and Coatings Technology, School of Industrial Technology, Universiti Sains Malaysia (USM-SIT), 11800 Penang, Malaysia; 4 Biological Resources and Post-harvest Division, Japan International Research Center for Agricultural Sciences (JIRCAS), Ohwashi, Tsukuba, Ibaraki 305-8686 Japan; 5 Faculty of Science, Kyushu University, Motooka, Nishi-ku, Fukuoka 819-0395 Japan; 6 Graduate School for International Development and Cooperation, Hiroshima University, Higashi-Hiroshima, Hiroshima 739-8529 Japan; 7 Present Address: Faculty of Bioengineering and Technology, Universiti Malaysia Kelantan, Jeli Campus, Locked Bag 100, 16700 Jeli, Kelantan Malaysia; 8 Present Address: Biological Resources and Post-harvest Division, Japan International Research Center for Agricultural Sciences (JIRCAS), Ohwashi, Tsukuba, Ibaraki 305-8686 Japan

**Keywords:** Plant sciences, Plant ecology

## Abstract

Oil palm is an important crop for global vegetable oil production, and is widely grown in the humid tropical regions of Southeast Asia. Projected future climate change may well threaten palm oil production. However, oil palm plantations currently produce large amounts of unutilised biological waste. Oil palm stems – which comprise two-thirds of the waste - are especially relevant because they can contain high levels of non-structural carbohydrates (NSC) that can serve as feedstock for biorefineries. The NSC in stem are also considered a potent buffer to source-sink imbalances. In the present study, we monitored stem NSC levels and female reproductive growth. We then applied convergent cross mapping (CCM) to assess the causal relationship between the time-series. Mutual causal relationships between female reproductive growth and the stem NSC were detected, with the exception of a relationship between female reproductive organ growth and starch levels. The NSC levels were also influenced by long-term cumulative temperature, with the relationship showing a seven-month time lag. The dynamic between NSC levels and long-term cumulative rainfall showed a shorter time lag. The lower temperatures and higher cumulative rainfall observed from October to December identify this as a period with maximum stem NSC stocks.

## Introduction

The global production of vegetable oil is constantly growing and is currently estimated at 199 million tonnes per year. The oil palm industry accounts for a considerable part of total vegetable oil production, with palm oil and palm kernel contributing 70 million and 9 million tonnes, respectively, in 2017/2018^1^. The average annual yield of palm oil from one hectare is about 3.7 tonnes, which is almost 10 times higher than that of other major oil crops^2^. Interestingly, 85% of all palm oil production is confined to a restricted area that is primarily within the countries of Indonesia and Malaysia. In this way, the uneven distribution of oil palm cultivation areas makes plants vulnerable to the unfavourable conditions caused by climate anomalies. For example, El Niño events in the Pacific Ocean can lead to reduce rainfall and increase air temperatures across oil palm plantation areas in Southeast Asia; these changes can lead to high levels of water stress among oil palm trees^3^. Crude palm oil (CPO) production is directly affected by the production of fresh fruit bunches (FFB), as FFB can neither be preserved nor sent to oil palm mills for processing after harvesting. There is a clear annual rhythm for CPO production in Peninsular Malaysia, with minimal production occurring from January to March and maximal production taking place between August and November^4^. This rhythm is assumed to be controlled by photoperiod response within a phytomer^5^, and is additionally influenced by environmental fluctuations mainly caused by El Niño – Southern Oscillation (ENSO)^3^.

A considerable proportion of the biomass residues from palm oil production has recently been identified as a potential source of renewable energy^6^. For each kilogram of palm oil, roughly another four kilograms of dry biomass are produced, of which approximately one-third is from fresh fruit bunch (FFB) waste and the other two-thirds from trunk and frond material^7,8^. Oil palm stem also contributes a large proportion of the waste, and its sap contains approximately 11% soluble sugars^9,10^. The non-structural carbohydrates (NSC), including soluble sugars in the stem, can be used as a substrate in bioreactors^6,11^, and using NSC is less costly than using structural carbohydrates^9,11,12^. However, the mechanisms through which NSCs accumulate in the stem have not yet been well documented.

The NSC in oil palm stem serve as the main buffer against fluctuations in carbon assimilation rates under source-sink imbalances^13^, and the production and storage of NSC are affected by various abiotic and biotic factors^14^. Limited resources and abiotic stresses have led to relatively low phenotypic plasticity regard to plant architecture or morphology; as such, the female reproductive organ (FRO) can be affected by climate variability through various reproductive processes, e.g. differentiation of inflorescence primordia, bunch abortion and delay of fruit bunch growth^13,15^. Therefore, it is necessary to investigate how environmental conditions and biotic factors influence oil palm phenotypes, which can be followed through reproductive growth and NSC levels in the stem.

Here, we monitored reproductive growth and NSC levels in oil palm stems for 3.5 years and applied recently developed convergent cross mapping^16^ to identify causal relationships between measurements from the stem and various abiotic factors. These findings should contribute to the stable production of palm oil as well as the utilisation of NSC from the stem in downstream applications.

## Results

### Time series fluctuations of focal biological traits and meteorology

#### Female reproductive organ (FRO)

**Figure 1:**
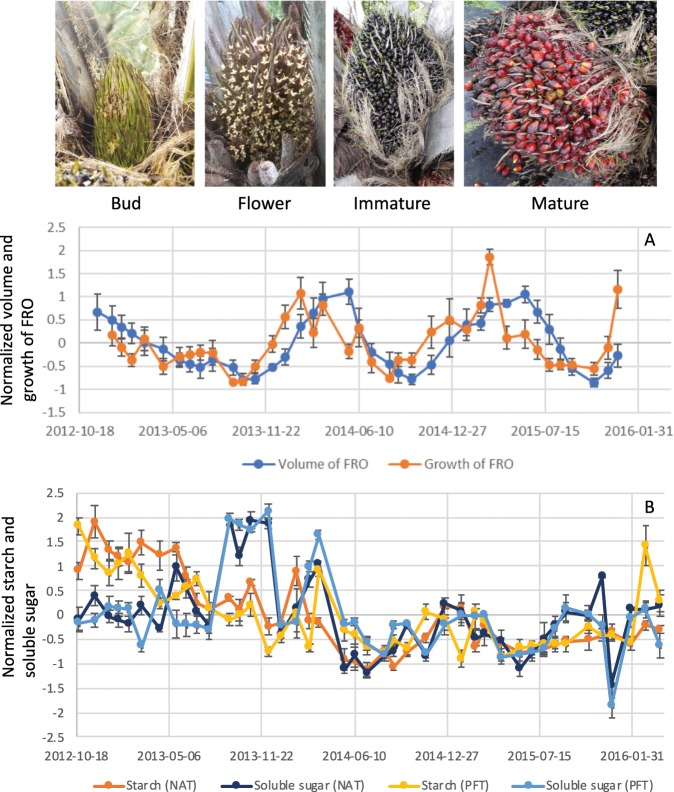
Time-series fluctuations of female reproductive growth in non-artificial treatment (NAT) trees (**A**) and non-structural carbohydrate of NAT and pruning female reproductive organs treatment (PFT) trees (NSC; **B**) during the study duration. Growth stages were defined according to the photos in the figure. The starches and soluble sugars classified as NSC were separately analysed.

The FRO volume showed two peaks during the observation period, in June 2014 and June 2015. In June, many fruit bunches matured and then dropped; therefore, FRO volume decreased until new buds emerged in the axil of each frond (Fig. 1). However, the development of all FROs was not synchronised. The increase in FRO volume was relatively larger from the bud to the immature stages; therefore, the increase in FRO growth preceded the increase in FRO volume (Fig. 1).

#### NSCs in stem

Although time series fluctuations of NSC did not show obvious seasonality, four peaks - November 2012, October 2013, January 2014, and December 2014 - were detected in starch content in non-artificial treatment (NAT) trees. The starch content in February 2016 was also slightly higher than what was observed for the surrounding sampling dates, whereas pruning FROs treatment (PFT) trees showed higher peaks at the sampling date. There was a small increase in starch content through the end to the beginning of the year. On the other hand, although soluble sugars in the stem did not show obvious seasonality, the highest and lowest peaks were detected from September to December 2013 and December 2015 in the both treatments, respectively (Fig. 1).

#### Meteorology

**Figure 2:**
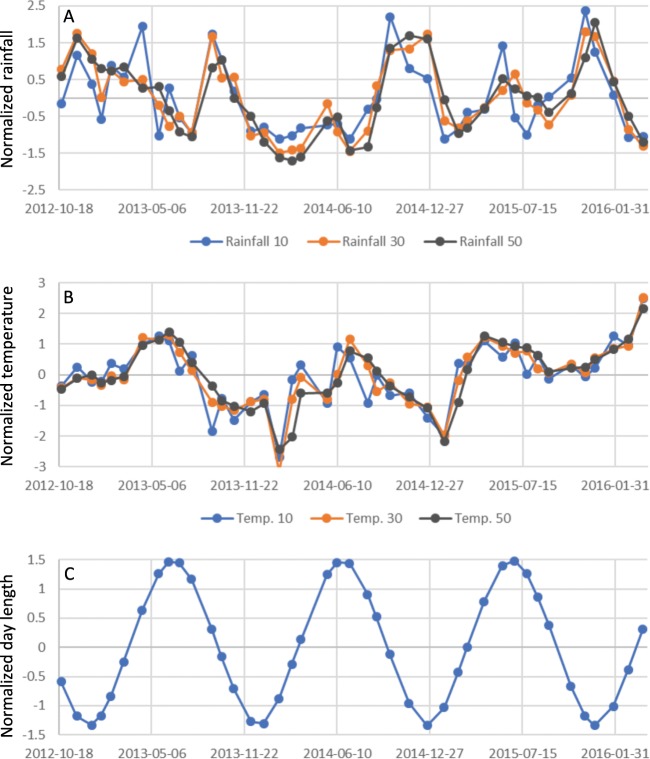
Time-series fluctuations of the studied environmental factors, cumulative rainfall (**A**) and cumulative temperature (**B**). Although cumulative days at 10, 30 and 50 are representatively shown in the figure, cumulative days from 1 to 60 were used in the analyses. Day length oscillation is also shown (**C**).

During the study period, a strong drought was recorded from December 2013 to June 2014. Annually, the study site received the highest rainfall from September to December (Fig. 2). The maximum rainfall per day was recorded on October 4^th^ 2013 (57.5 mm). Annual rainfall at the site was over 2,000 mm, but in 2014 it was less than 2,000 mm due to the strong drought. The highest and lowest temperatures during the study period were 30.9 °C and 23.9 °C, respectively. Even though there was a small range of temperatures, seasonality of temperature was nevertheless observed, namely, the highest and lowest temperatures were recorded around May-June and January-February, respectively (Fig. 2). This pattern is similar to day length fluctuations at the site (Fig. 2).

### Causal relationships between biological traits

**Figure 3:**
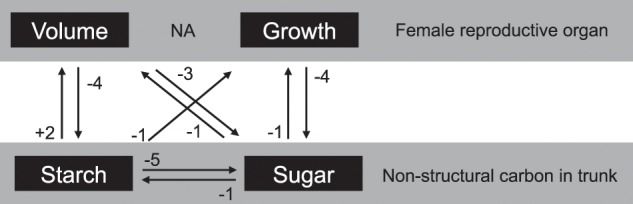
CCM analyses of the causality between biological time-series. Arrows depict relationships that are significantly supported by the 95% upper surrogate. Numbers beside the allows represent time lags.

The causal relationship of biological traits, that is, between NSC in the stem and female reproductive organ characteristics (total volume and growth from the previous month), were mutually evaluated. A unidirectional causal relationship was identified between the starch content in the stem and the growth of FRO with a −1 month time lag, and this was supported by a 95% upper boundary of the surrogate and convergence of predictive skill (*ρ*) (Table 1, Fig. 3 and Supplemental Table 1). Multiple mutual causal relationships were also identified between the two biological time series (Table 1 and Fig. 3). Starch content in the stem and FRO volume showed a mutual causal relationship that was supported by a 95% upper boundary of the surrogate and convergence of predictive skill (*ρ*). However, large differences in time lag were identified (the time lag for the relationship from FRO volume to starch levels in the stem was −4 months, and +2 months in the opposite direction; Table 1 and Fig. 3). In the relationship between soluble sugar levels and FRO volume, the differences in time lag were relatively smaller, totaling two or three months. In the causal relationship that described how soluble sugar levels affect FRO volume, a −1 month lag was seen, with a -3 month lag in the opposite direction. In the causal relationship of soluble sugar levels and FRO growth, a −1 month lag was identified, with a −4 month lag observed for the opposite direction (Table 1 and Fig. 3). Among all the relationships between two variables, the highest predictive skill (*ρ*) was identified for the causal relationship from FRO volume to soluble sugar levels in the stem (Table 1).

**Table 1:** Estimated predictive skills (*ρ*) between observed biological traits using CCM. Time-series of a trait as target was predicted from another time-series as library.

Library	Time-series data	Time lag												
	Target	−6	−5	−4	−3	−2	−1	0	+1	+2	+3	+4	+5	+6
FRO volume	Starch in stem	−0.022	0.052	0.106	0.123	0.156	0.228	*0.308*	*0.367*	***0.378***	*0.344*	0.262	0.145	0.046
Starch in stem	FRO volume	*0.351*	*0.340*	***0.360***	*0.320*	0.230	0.132	0.106	0.142	0.137	0.066	0.018	−0.026	−0.052
FRO growth	Starch in stem	0.046	0.037	0.074	0.111	0.232	***0.333***	*0.309*	*0.314*	*0.330*	*0.307*	0.277	0.199	0.092
Starch in stem	FRO growth	0.303	0.292	0.178	0.102	0.122	0.132	0.143	0.099	0.056	0.067	−0.029	0.027	0.090
FRO volume	Soluble sugar in stem	0.249	*0.391*	*0.432*	*0.448*	*0.448*	***0.458***	*0.361*	*0.331*	0.304	0.240	0.155	0.089	0.047
Soluble sugar in stem	FRO volume	*0.507*	*0.519*	*0.544*	***0.588***	*0.569*	*0.460*	*0.286*	0.171	0.122	0.148	0.151	0.058	−0.021
FRO growth	Soluble sugar in stem	0.125	0.124	0.188	*0.258*	*0.304*	***0.464***	*0.347*	*0.353*	*0.299*	*0.310*	*0.244*	0.162	0.049
Soluble sugar in stem	FRO growth	*0.403*	*0.382*	***0.409***	*0.322*	*0.248*	0.105	0.145	0.161	0.131	0.087	−0.030	−0.063	−0.080
Starch in stem	Soluble sugar in stem	*0.516*	*0.526*	*0.516*	*0.522*	*0.530*	***0.531***	*0.490*	*0.392*	*0.348*	*0.405*	0.299	0.232	0.124
Soluble sugar in stem	Starch in stem	*0.563*	***0.577***	*0.568*	*0.562*	*0.548*	*0.546*	*0.514*	*0.385*	0.265	0.160	0.111	0.137	0.049

^1^Signficance of predictive skill (*ρ*) was evaluated by 95% upper boundary of twin surrogate (Thiel *et al*. 2006, Ushio *et al*. 2018) which was showed by italic and bold expressed the largest predictive skill among those of time lag.

A mutual causal relationship between soluble sugars and starch in the stem was also detected. The maximum predictive skill was detected at a −1 month time lag for the causal direction from soluble sugars to starch, with a −5 month time lag identified for the opposite direction (Table 1, Fig. 3 and Supplemental Table 1).

### Causal relationships from meteorological environments to biological traits

We applied the daily cumulative temperature (CT) and rainfall (CR) in a range from observation day 1 up to 60 days. We did not evaluate the causal relationships of the CT and the CR for more than 60 days, because there is a tendency to lose the non-linearity for the CT and CR over longer cumulative periods, which was confirmed through the ‘smap’ function of rEDM.

**Figure 4:**
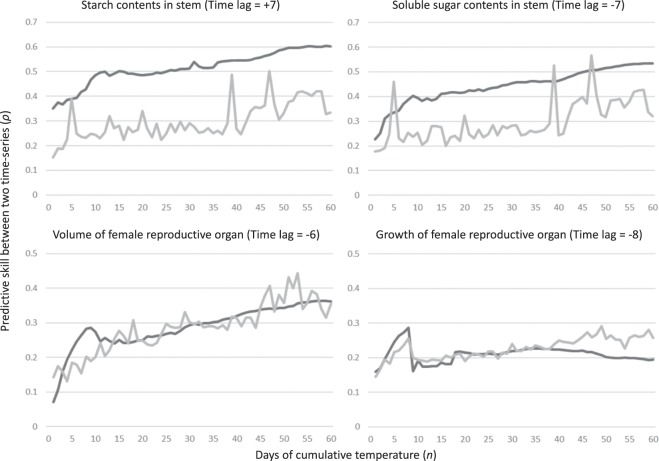
Predictive skill (*ρ*; solid black line) values, along with the 95% upper limit of the surrogate (grey solid line), of causality from cumulative temperature to biological time-series, starch contents in the stem, soluble sugar contents in the stem, volume of the female reproductive organ and growth of the female reproductive organ.

**Figure 5:**
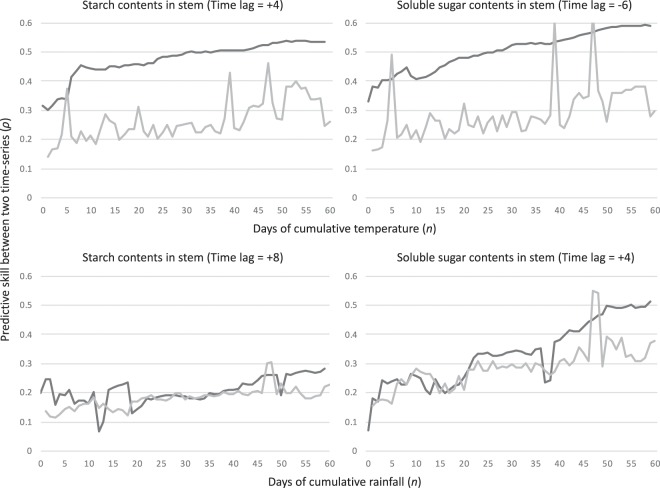
Predictive skill (*ρ*; solid black line) values, along with the 95% upper limit of the surrogate (grey solid line), of causality from cumulative temperature to starch contents and soluble sugar contents in the pruning female reproductive organs treatment (PFT) stems (upper), and these of causality from cumulative rainfall to starch contents and soluble sugar contents in the PFT stems (lower).

Regarding cumulative temperature, a significant and strong causal relationship between this parameter and both starch and soluble sugars in the stem was detected for all cumulative periods. The maximum predictive skill (*ρ*) was observed at 55 cumulative days with a +7 month time lag for the starch, and at 58 cumulative days with a −7 month time lag for the soluble sugars in the stem of NAT (Fig. 4). These results were confirmed by PFT trees, showing that the maximum predictive skill (*ρ*) was observed at 55 cumulative days with a +4 month time lag for the starch, and at 59 cumulative days with a −6 month time lag for the soluble sugars (Fig. 5). Although time lag of starch in PFT tree showed a larger difference from the lag of NAT trees (three months), predictive skill (*ρ*) of +7 month time lag also showed higher predictive skill (*ρ*) (Supplemental Table 3). On the other hand, the causal relationship from the CT to FRO was relatively weak. Although the maximum predictive skill (*ρ*) from the CT to the FRO volume was detected at 59 days with a −6 month time lag, half of the cumulative days in the long-term (*n* = 41 to 60) showed lower predictive skill (*ρ*) than the 95% surrogate. In the short-term, the predictive skill showed a peak at nine days, and its significance was supported by a 95% upper bound of the surrogate (Fig. 4). FRO growth showed a similar pattern as FRO volume, however, the predictive skill (*ρ*) was not significant in the long-term. A peak of predictive skill (*ρ*) from 6–8 days was slightly larger than the 95% upper bound of the surrogate with a −8 month time lag (Fig. 4). Overall, the causal relationship from the temperature to the focal biological traits confirmed that temperature is one of the main factors influencing NSC in the oil palm stem, but temperature only exerts a weak effect on the FRO. Convergence of predictive skill (*ρ*) was confirmed in all significant causal relationships from the CT to the biological traits. (Supplemental Table 2).

**Figure 6:**
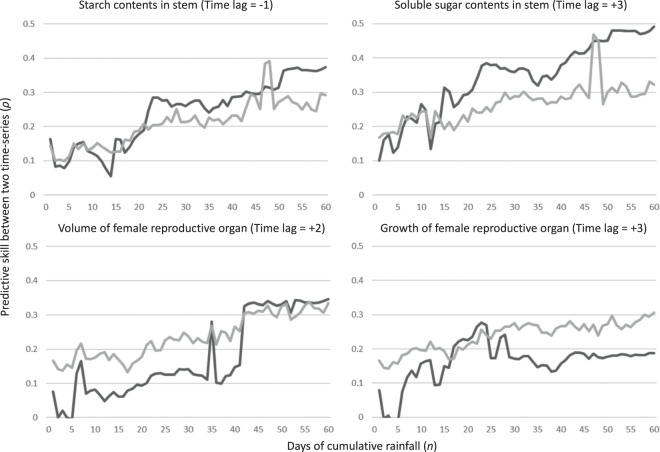
Predictive skill (*ρ*; solid black line) values, along with the 95% upper limit of surrogate (grey solid line), of causality from cumulative rainfall to biological time-series, starch contents in the stem, soluble sugar contents in the stem, volume of the female reproductive organ and growth of the female reproductive organ.

When the effects of cumulative rainfall were assessed, the starch and soluble sugars in the NAT stem showed weak and strong causal relationships with this abiotic factor in the mid- and long-term cumulative days with a −1 and a +3 month time lag, respectively. The maximum predictive skill (*ρ*) was detected at 60 cumulative days for both starch and soluble sugars (Fig. 6). This trend was also confirmed in PFT stems. There was causal relationship from rainfall to starch in PFT stems, although it was very weak and the selected time lags with a maximum predictive skill were different between NAT and PFT in starch (Fig. 5). The FRO volume only showed significant predictive skill (*ρ*) in the long-term cumulative days with a +2 month time lag. In contrast, FRO growth only showed significant predictive skill (*ρ*) in the mid-term cumulative days. The maximum predictive skill (*ρ*) values for FRO volume and growth were detected at 60 and 23 cumulative days, respectively (Fig. 6). The convergence of predictive skill (*ρ*) was confirmed for all significant causal relationships from the CR to the biological traits (Supplemental Table 2).

## Discussion

### Causal relationship between NSC in stem and female reproductive growth

The two biological traits investigated in this study were mutually related to each other except for the causal relationship from FRO growth to starch amounts in the stem. In general, most of a plant’s carbohydrates are produced through photosynthesis in leaves, and are then translocated based on sink location^17^. In oil palm, Corley *et al*.^18^ showed that dry weight of reproductive bunches per palm per year is equivalent as dry weight of vegetative parts per palm per year, which indicates that substantial large allocation of photosynthesis assimilates into reproductive bunches. Further, fraction of male inflorescences is smaller than female inflorescences (less than ca. 30%)^19^, and the male inflorescence has an average weight of just 2 kg and does not remain for a long period on the palm tree, progressing to senescence in about one month after anthesis, and liberating the oil palm tree from further energy demand^20^. Therefore, allocation of photosynthesis assimilates to male reproductive growth is relatively much smaller than it to female reproductive growth, which indicates that FRO is main sink in oil palm. NSC in the stem is considered a major physiological buffer that guards against seasonal source-sink imbalances^5,13^, their compensatory function has been shown to surpass what was observed in coconut trees^21^. Our analyses of the causal relationships between important biological traits also identify NSC levels in the stem as a physiological buffer of source-sink imbalances. Artificial sink limitation by complete fruit pruning was shown to accelerate development rate, increasing phytomer, leaf and inflorescence numbers as well as assimilate storage – mainly starch – in the stem. In contrast, glucose, which was identified as the most abundant NSC in control oil palms, decreased^22^. Although our experiment in NAT did not involve artificial sink limitation, we observed three times during the observation period at which fresh fruit bunches decreased. At these times, we observed a distinct rise in soluble sugar content and an unclear rise in starch content in the stem (Fig. 1). The highest predictive skill (*ρ*) was observed in the causal relationship from FRO volume to soluble sugar content in the stem with −3-month time lag, which surpassed the predictive skill (*ρ*) of the causal relationship from FRO growth to soluble sugar content in the stem with a −4 month time lag. The relationship in the opposite direction, from soluble sugar content to both the volume and growth of FRO, also showed significant predictive skill (*ρ*); however, the predictive skills (*ρ*) were lower and included less time lag (−1-month; Table 1). These results support that soluble sugar acts as one of the main buffers against source-sink imbalances. On the other hand, our CCM analyses showed lower predictive skill (*ρ*) estimates for starch in the stem and no significant predictive skill (*ρ*) values were detected for the causal relationship from FRO growth to starch in the stem (Table 1). Although Legros, *et al*.^22^ found starch to be the main assimilate in the stem under sink-limited conditions, the weak causal relationship, and considerable time lag, between FRO volume and starch levels in the stem might be explained by an indirect relationship between the two traits through starch biosynthesis from soluble sugars in the stem. This idea is supported by the significant and large predictive skill (*ρ*) values for the causal relationship from soluble sugars to starch in the stem (the values are also significant in the opposite direction, from starch to soluble sugar in the stem; Table 1).

### Environmental regulation of female reproductive growth fluctuations and source-sink imbalances

**Figure 7:**
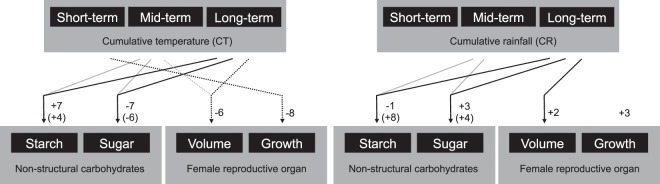
CCM results regarding the causality from environmental time-series (cumulative temperature and rainfall) to biological time-series. Allows depict relationships that are significantly supported by the 95% upper surrogate. Short-, mid- and long-term were defined as 1–20, 21–40, and 41–60 cumulative days, respectively. Solid black and solid grey arrows represent the highest, second highest predictive skill (*ρ*) values, respectively, whose significance was detected in majority of cumulative days. The dotted arrow represents the highest predictive skill (*ρ*) values but non-significant *ρ* detected in more than five cumulative days within each term. Numbers and numbers in parenthesis beside the allow represent time lags of NAT and PFT, respectively.

It has been known that an oil palm’s yield of fresh fruit bunches (FFB) shows seasonal rhythm^3,5,23^. This is especially relevant when sea-surface temperatures change – usually as a result of El Niño events in the tropical Pacific Ocean – leading to reduced rainfall and increased air temperatures, which together contribute to high water stress levels for oil palm trees. These unfavourable environments enhance male inflorescence production, which is one the reasons underlying reductions in FFB yield^24^. In our experiment, we monitored the volume and growth of FRO and observed two major peaks in the middle of the observation period (Fig. 1). According to the CCM results, the largest significant predictive skill (*ρ*) of causality from rainfall to FRO volume was detected in the longer cumulative days with a +2 month time lag (Fig. 6). The largest significant predictive skill (*ρ*) for the causal relationship from rainfall to FRO growth was in the middle cumulative duration with a +3 month time lag. This does not agree with Legros, *et al*.^5^ estimated that plants are the most sensitive to drought effects 29 months before bunch maturity. Although it was impossible to verify longer time lags due to our relatively short observation period, our CCM results show much shorter time lags for the causality from rainfall to reproductive growth. Generally, inflorescence development in mature oil palm requires around 2–3 years^25^, with bunch number per stem, bunch weight and the oil component as the main factors influencing oil palm reproductive growth. The bunch number per stem is largely affected by sex determination during inflorescence development, which occurs approximately 22 months before flower maturity in adult oil palms^5,25,26^. This time lag between sex determination and flower/fruit maturity is considerably longer than the time lag detected in the CCM analysis of causality from rainfall to both volume and growth of the reproductive organ. The CCM analysis may have detected a causal relationship with a shorter time lag from rainfall to fruit maturity, which showed two obvious peaks in May 2014 and 2015 (volume) and February 2014 and March 2015 (growth) (Fig. 1). The incomplete synchronisation of fruit maturity caused a rapid reduction in FRO volume and growth after the peaks (Fig. 1). This timing should be synchronised with rainfall fluctuation with short time lags (Figs. 1, 3 and 6). On the other hand, the relationship between long-term cumulative temperature and FRO volume showed slightly significant predictive skill (*ρ*) with a maximum value at a relatively large time lag (−6-month). The significance (i.e. supported by 95% of upper surrogates) was not consistent among all of the cumulative temperature periods (Fig. 4). Regarding FRO growth, the only significant predictive skill (*ρ*) values among cumulative temperature periods were for the peaks observed for short cumulative days (Fig. 4). These findings suggest that reproductive growth is not strongly regulated by temperature (Fig. 7).

In NSC levels, soluble sugar in the stem were strongly regulated by longer cumulative periods of both rainfall and temperature, whereas starch in the stem was strongly regulated only by mid and longer cumulative temperature (Figs. 4, 5 and 6), which provides additional evidence for the role of these compound in buffering against source-sink imbalances^13^. The larger predictive skill (*ρ*) value seen for temperature in the both NAT and PFT supports that longer periods of a sustained temperature are more effective at regulating stem NSC levels than long periods of rainfall (Figs. 4, 5 and 6). However, rainfall caused an immediate response (shorter time lags in NPT) in terms of NSC reserve fluctuation relative to temperature (Figs. 4 and 6). Especially, starch in stem is possibly main regulator to control source sink imbalances caused by rainfall, which has been evidenced by large difference of lag time of causal relationship from long-term cumulative rainfall to starch between NAT and PFT (Fig. 7 and Supplemental Table 3). As oil palms originate from equatorial humid environments^15^, these plants are highly sensitive to water deficits, which should stimulate a quick response of NSC levels – either through physiological or morphological mechanisms linked to CO_2_ assimilation or reproductive growth – to buffer against source-sink imbalances^13^. Because the above ground vegetative growth of oil palm is synchronized with environments with less time-lag^13^, it is possible that the source-sink imbalances are regulated by the balance of CO_2_ assimilation level and reproductive growth. On the other hand, there are several potential reasons for why long periods of constant temperature impact NSC levels in the stem. First, thermoperiodism can be compared with photoperiod (Fig. 2). Plants in equatorial environments are able to sense even small changes in day and night length by the circadian clock^27^. Legros, *et al*.^5^ insisted that photoperiodism is the baseline for reproductive growth fluctuations, and this may partly explain the causality from photoperiod (long cumulative days of temperature) to NSC levels in the stem. However, this effect should not be considered as a main regulatory element because of the weak causality from long cumulative days of temperature to female reproductive growth (Fig. 4). It has been reported that the circadian clock can increase photosynthesis in model plant species such as *Arabidopsis thaliana*^28,29^. Subsequent products of photosynthesis are then consumed or stored through starch production/consumption, which is also under circadian control^30^. In this way, it is possible that NSC reserves in the oil palm stem are under circadian control, which may explain the effect that long periods of sustained temperature have on starch and soluble sugar contents. This mechanism was evidenced by that the causality from long cumulative days of temperature to starch was well conserved, when we removed the effect of female reproduction by pruning FROs (Fig. 5). On the other hand, the CCM analysis showed the largest predictive skill (*ρ*) value to occur at a time lag of seven months for the relationship from long cumulative days of temperature to starch levels in the NAT stem (the maximum *ρ* value for the relationship between temperature and soluble sugar levels in the NAT stem was seen at −7 months; Fig. 4). Slight oscillations in temperature and day length occur even in the low latitude tropical area (Fig. 2). A time lag of over half a year reflects completely opposite oscillations between NSC reserves and long cumulative days of temperature. Unfortunately, the factors that influence these conflicting oscillations remain unknown, which shall be studied in future.

### Linking NSC levels in the stem to the provision of substrate for biorefinery

Circadian rhythms and rainfall both regulate the oscillation of NSC levels in oil palm stems. Changes in the female reproductive organ weakly regulate starch levels but strongly regulate soluble sugar levels. NSC (both starch and soluble sugar) from the oil palm stem constitute an abundant and promising resource for the bioenergy sector because: 1) the stem is normally felled once the plant reaches 25 years of age due to decreasing fruit bunch production, after which the stem is discarded without any energy capture; and 2) the NSC in stem are available for cheaper than structural carbohydrates such as cellulose and lignin^9,11,12^. Our results provide information that can used to time fellings to periods that will maximise the NSC resources in the stem. For example, the period from October to December showed lower cumulative temperatures (shorter day length) and higher cumulative rainfall (Fig. 2), along with slower development of the female reproductive organ (Fig. 1). Performing the felling during this time should increase the probability that oil palms have significant NSC reserves in the stem.

## Materials and Methods

### Study site, experimental plant and measurement of reproductive growth

The experiment was carried out between October 23^rd^ 2012 and March 31^st^ 2016 on four oil palm trees (cultivar *tenera*, more than 25 years old) planted in a campus of Universiti Sains Malaysia (USM), Penang state of Malaysia (5°08′37″N, 100°29′39″E). Four studied palm trees were under no artificial treatments (NAT trees), such as fruit pruning, defoliation and fertilization during the experimental period. On the other hand, a treatment to prune FROs had been performed since April 15^th^ 2013 until the end of the experimental period for the other four studied palm trees (PFT trees). During the experimental period, female reproductive growth was recorded monthly for the NAT trees (152 time points from 38 time points with 4 palm trees). Once the rachis meristem emerged between the base of the two fronds, female reproductive growth was categorised into four stages of development, namely, buds, flowers, and immature and mature fruits (Fig. 1). The three-dimensional size (*a*, width; *b*, depth; and *c*, height) of the female reproductive organ (FRO) was measured monthly, and its volume was calculated as an ellipsoid. Sexual determination of the reproductive organ at the bud stage was performed retroactively based on observations after flowering. For each experimental tree, the total volume of the FROs and female reproductive growth rate per day from time *t *− *1* to point *t* were calculated using the following equations,

$${V}_{t}=\mathop{\sum }\limits_{i=1}^{m}\frac{4}{3}\pi {a}_{i}{b}_{i}{c}_{i}\,{\rm{and}}\,{G}_{t}=\frac{{V}_{t}-{V}_{t-1}}{{d}_{t}-{d}_{t-1}},\,{\rm{respectively}}.$$

where *a*, *b* and *c* represent the three-dimensional measurements of the *i*th reproductive organ of each studied tree, *V*_*t*_ is the total cubic volume of a studied palm tree at time point *t*, *G*_*t*_ is the monthly growth in volume from time point *t* − 1 to *t* and *d* is the number of days between time points *t* and *t* − *1*. The comparison between *V*_*t*_ and wet weight of 117 pruned FROs from the PFT trees in the study site showed significant linear relationship (Data not shown).

### Non-structural carbohydrates (NSC) in the oil palm stem

Stem core samples were collected from three height levels (top, middle and bottom) of the four NAT and four PFT stems using increment borers at every time point (168 time points from 42 time points with 4 trees of each treatment). The woody core tissue was immediately transferred to the laboratory at a low temperature and stored in the freezer (−20 °C) prior to the experiment. The starch and soluble sugars that can be classified as NSC were separately extracted from woody tissue; unspecified sites of core sample were used in the extraction process because vascular bundles are irregularly distributed over the stem cross section (atactostele) in Arecaceae^31^.

### Measuring starch content in oil palm stems

The enzyme assay was performed using commercial enzymes adopted from the total assay procedure method (AOAC Method 996.11/ AACC Method 76.13) from Megazyme (Megazyme Inc., Bray, Ireland). Thermostable α-amylase (3,000 U/mL) was diluted in MOPS buffer (Sigma-Aldrich, St. Louis, MO) at pH 7.0 followed by an incubation in C_2_H_3_NaO_2_ buffer (200 mM) at pH 4.5 and amyloglucosidase concentrate (3,300 U/mL). Total starch content was determined by the enzymatic hydrolysis of 100 mg of fine powder from each sampled stem core. The samples were hydrolysed using 3 mL of thermostable α-amylase to extract maltodextrin from starch at 100 °C over 6 min (vortex at 2, 4 and 6 min intervals). The slurry samples were incubated at 50 °C in a water bath with 4 mL of C_2_H_3_NaO_2_ buffer, followed by 0.1 mL of amyloglucosidase for 30 min to hydrolyse maltodextrin to glucose. The amount of glucose present was measured using an UV-VIS spectrophotometer at 510 nm (Perkin Elmer Lamda 35 UV/VIS Spectrophotometer, Perkin Elmer, MA, USA).

### Measuring soluble sugar content in oil palm stems

Approximately 120 mg of core powder from each sampled tree was weighed and soaked in 10 mL of water. Slurry samples were then vortexed to ensure that all free sugars had been extracted into water, followed by incubation in boiling water (100 °C) for 30 min with mixing. After the samples were cooled to room temperature, 1 mL of supernatant was transferred, diluted two times, and mixed vigorously. After centrifuging at 10,000 rpm for 10 min, the supernatant was filtered using a 0.45 μm PTFE column. The filtrate then underwent sugar analysis using HPLC (SIL-20A/20AC; Shimadzu, Kyoto, Japan) and a CarboSep CHO-620 carbohydrate analytical column. The employed mobile phase was double distilled water at a flow rate of 0.4 mL/min, and the column temperature was 80 °C. A CAR 10 carbohydrate kit (Sigma-Aldrich) was used as a standard for the calibration curve.

### Meteorological data

Daily average temperature was estimated from daily maximum and minimum temperatures collected from the meteorological station at JKR Bagan Serai office (5°07′54″N, 100°37′48″E), about 15.3 km away from the study site (data were provided by Malaysian meteorological department). Daily maximum temperature data were missing on August 23^rd^ and November 12^th^ 2013; May 12^th^ to 27^th^, August 21^st^, October 28^th^, and November 29^th^ 2015; and February 23^rd^ and March 5^th^ 2016. Daily minimum temperature data were missing on July 23^rd^ and August 17^th^ 2013. We calculated the average temperature using temperatures before and after the missing time points to fill the blanks. Cumulative temperatures (*CT*_*n*_) were calculated by summing the daily average temperatures during past *n* days from time point *t* (*n* ranges from 1 to 60 days) as follows,

$$C{T}_{n}=\mathop{\sum }\limits_{j=0}^{n-1}{T}_{j}.$$

where *T* is the daily average temperature on the *j*th day from time point *t*. Daily rainfall data were collected at Bukit Panchor hydrological station, which is under the jurisdiction of the Department of Irrigation and Drainage, Ministry of Water Land and Natural Resources. The station (5°09′38″N, 100°32′06″E) is about 5.1 km away from the study site. Cumulative rainfall (*CR*_*n*_) was also calculated by summing the daily rainfall during the past *n* days from time point *t* (*n* ranges from 1 to 60 days) as follows,

$$C{R}_{n}=\mathop{\sum }\limits_{j=0}^{n-1}{R}_{j}.$$

Here, *R* is the daily rainfall on the *j*th day from time point *t*. Day length (*DL*_*t*_) was calculated from times of sunrise and sunset at the study site at time point *t*, and is used for the convergent cross mapping analysis.

### Detection of causal relationships

The convergent cross mapping (CCM) method^16^ was applied to identify mutual causal relationships between two biological time series variables, i.e. the female reproductive organs (volume at time point *t* and growth from time point *t –* 1 to *t*) and the assimilate products in the NAT stem at time point *t*. Although NSC levels follow a gradient, with higher levels at the top and lower levels at the bottom of the stem^13^, average starch and soluble sugar contents from the top, middle and bottom of a single experimental tree were calculated for the following data analysis to observe trends in NSC levels across the whole stem. Causal relationships between the meteorological and biological time series variables were also evaluated by CCM using NAT and PFT trees. As biological replicates from the four monitored trees can be considered to be equivalent, a composite time series approach employing dewdrop regression was applied to the CCM for each treatment (NAT and PFT)^32^. After checking the non-linearity of all the biological and meteorological time series by S-map analysis of rEDM^33,34^, an embedding dimension (*E*) at maximum predictive skill (*ρ*) was selected as the optimal, and we conducted cross-mapping between data from two time series from 2 to 8 *E* with a fixed library size (all 168 time series points) and −1 time lag (*tp*) condition using the ‘ccm’ function in rEDM^34–36^. The maximum *E* was applied to subsequent cross-mapping between two time series data to estimate the predictive skill (*ρ*). Next, the significance of the predictive skill was evaluated by converging predictive skill (*ρ*) with an increment of the library size and 95% of upper surrogate - which was randomly generated 1,000 times in rEDM using the twin surrogate method^37,38^. The twin surrogate method can avoid false positive causality because it considers seasonality between two focal time-series. These time-series data set used for CCM was deposited to Dryad (Accession No is 10.5061/dryad.rxwdbrv4c).

## Supplementary information

Supplemental Tables 1-3.

## Data Availability

Time series data on NSC in stem (starch and sugar content), reproductive traits and meteorology (cumulative temperature and rainfall) are deposited to Dryad. Accession No. is 10.5061/dryad.rxwdbrv4c.
