# Supplementary material for: Small temperature variations are a key regulator of reproductive growth and assimilate storage in oil palm (Elaeis guineensis)
Source: Sci Rep. 2020 Jan 20;10:650. doi: 10.1038/s41598-019-57170-8 (PMC6971258; doi:10.1038/s41598-019-57170-8)
Supplement: Supplementary file 1 — Supplemental Tables 1-3. [file 41598_2019_57170_MOESM1_ESM.pdf]

Title: Supplemental Table 1. Estimated predictive skills (ρ) and standard deviation (SD) of ρ at every library size between observed biological traits using CCM. Combinations of the two biological traits with significant ρ were shown in the table.

Paper Title: Small temperature variations are a key regulator of reproductive growth and assimilate storage in oil palm (Elaeis guineensis)

Author list: Naoki Tani, Zubaidah Aimi Abdul Hamid, Natra Joseph, Othman Sulaiman, Rokiah Hashim, Takamitsu Arai, Akiko Satake, Toshiaki Kondo, Akihiko Kosugi

Supplemental Table 1. Estimated predictive skills (ρ) and standard deviation (SD) of ρ *at every library size* between observed biological traits using CCM. Combinations of the two biological traits with significant ρ were shown in the table

Time-series data		CCM result				
Library	Target	optimal *E*	selected *tp*	library size	*ρ*	SD of *ρ*
FRO volume	Starch in stem	8	2	8	0.249	0.164
		8	2	13	0.282	0.148
		8	2	18	0.319	0.130
		8	2	23	0.331	0.108
		8	2	28	0.360	0.089
		8	2	33	0.364	0.086
		8	2	38	0.378	0.067
Starch in stem	FRO volume	6	-4	6	0.170	0.200
		6	-4	11	0.239	0.159
		6	-4	16	0.281	0.128
		6	-4	21	0.311	0.105
		6	-4	26	0.333	0.110
		6	-4	31	0.340	0.107
		6	-4	36	0.355	0.091
		6	-4	41	0.360	0.087
FRO growth	Starch in stem	8	-1	8	0.175	0.185
		8	-1	13	0.252	0.149
		8	-1	18	0.291	0.137
		8	-1	23	0.315	0.109
		8	-1	28	0.321	0.098
		8	-1	33	0.320	0.098
		8	-1	38	0.333	0.081
FRO volume	Soluble sugar in stem	8	-1	8	0.279	0.182
		8	-1	13	0.372	0.139
		8	-1	18	0.403	0.127
		8	-1	23	0.418	0.104
		8	-1	28	0.448	0.077
		8	-1	33	0.449	0.082
		8	-1	38	0.458	0.069
Soluble sugar in stem	FRO volume	7	-3	7	0.352	0.190
		7	-3	12	0.482	0.139
		7	-3	17	0.525	0.101
		7	-3	22	0.546	0.088
		7	-3	27	0.558	0.079
		7	-3	32	0.570	0.074
		7	-3	37	0.571	0.075
		7	-3	42	0.588	0.059
FRO growth	Soluble sugar in stem	7	-1	7	0.290	0.180
		7	-1	12	0.391	0.151
		7	-1	17	0.419	0.124
		7	-1	22	0.423	0.115
		7	-1	27	0.454	0.088
		7	-1	32	0.450	0.085
		7	-1	37	0.460	0.077
		7	-1	42	0.464	0.065
Soluble sugar in stem	FRO growth	5	-4	5	0.193	0.217
		5	-4	10	0.284	0.185
		5	-4	15	0.339	0.152
		5	-4	20	0.368	0.118
		5	-4	25	0.372	0.116
		5	-4	30	0.388	0.100
		5	-4	35	0.399	0.085
		5	-4	40	0.409	0.079
Starch in stem	Soluble sugar in stem	8	-1	8	0.342	0.161
		8	-1	13	0.421	0.129
		8	-1	18	0.479	0.102
		8	-1	23	0.504	0.078
		8	-1	28	0.517	0.077
		8	-1	33	0.533	0.069
		8	-1	38	0.531	0.068
Soluble sugar in stem	Starch in stem	8	-5	8	0.416	0.123
		8	-5	13	0.498	0.079
		8	-5	18	0.535	0.058
		8	-5	23	0.555	0.049
		8	-5	28	0.572	0.043
		8	-5	33	0.572	0.043
		8	-5	38	0.577	0.039

Title: Supplemental Table 2. Supplemental Table 2. Estimated predictive skills (ρ) and standard deviation (SD) of ρ at every library size of causal relationship from meteorological to biological time-serieses on non-artificial treatment (NAT) using CCM..

Paper Title: Small temperature variations are a key regulator of reproductive growth and assimilate storage in oil palm (Elaeis guineensis)

Author list: Naoki Tani, Zubaidah Aimi Abdul Hamid, Natra Joseph, Othman Sulaiman, Rokiah Hashim, Takamitsu Arai, Akiko Satake, Toshiaki Kondo, Akihiko Kosugi

Supplemental Table 2. Estimated predictive skills (ρ) and standard deviation (SD) of ρ at every library size of causal relationship from meteorological to biological time-serieses on non-artificial treatment (NAT) using CCM.

Time-series data		CCM result				
Library	Target	optimal *E*	selected *tp*	library size	ρ	SD of ρ
Starch in stem	CT (59 days)	7	7	7	0.514	0.137
		7	7	12	0.570	0.091
		7	7	17	0.579	0.076
		7	7	22	0.585	0.061
		7	7	27	0.594	0.045
		7	7	32	0.598	0.042
		7	7	37	0.602	0.039
		7	7	42	0.603	0.039
Soluble sugar in stem	CT (58 days)	8	-7	8	0.327	0.165
		8	-7	13	0.430	0.123
		8	-7	18	0.470	0.115
		8	-7	23	0.502	0.086
		8	-7	28	0.509	0.079
		8	-7	33	0.530	0.067
		8	-7	38	0.533	0.073
FRO volume	CT (59 days)	8	-6	8	0.218	0.168
		8	-6	13	0.290	0.119
		8	-6	18	0.307	0.112
		8	-6	23	0.329	0.097
		8	-6	28	0.344	0.082
		8	-6	33	0.351	0.082
		8	-6	38	0.363	0.074
FRO growth	CT (8 days)	8	-8	8	0.145	0.175
		8	-8	13	0.205	0.149
		8	-8	18	0.233	0.137
		8	-8	23	0.260	0.124
		8	-8	28	0.279	0.114
		8	-8	33	0.272	0.109
		8	-8	38	0.287	0.088
Starch in stem	CR (60 days)	8	-1	8	0.201	0.183
		8	-1	13	0.237	0.140
		8	-1	18	0.266	0.142
		8	-1	23	0.318	0.136
		8	-1	28	0.340	0.109
		8	-1	33	0.358	0.103
		8	-1	38	0.374	0.093
Soluble sugar in stem	CR (60 days)	8	3	8	0.324	0.170
		8	3	13	0.411	0.151
		8	3	18	0.444	0.137
		8	3	23	0.476	0.119
		8	3	28	0.474	0.117
		8	3	33	0.483	0.095
		8	3	38	0.492	0.090
FRO volume	CR (60 days)	8	2	8	0.198	0.168
		8	2	13	0.231	0.169
		8	2	18	0.298	0.128
		8	2	23	0.313	0.131
		8	2	28	0.329	0.110
		8	2	33	0.340	0.091
		8	2	38	0.346	0.090
FRO growth	CR (23 days)	8	3	8	0.151	0.160
		8	3	13	0.186	0.153
		8	3	18	0.241	0.131
		8	3	23	0.250	0.126
		8	3	28	0.277	0.100
		8	3	33	0.277	0.103
		8	3	38	0.276	0.096

Title: Supplemental Table 3. Estimated predictive skills (ρ) and standard deviation (SD) of ρ at every library size of causal relationship from meteorological to biological time-serieses on pruning female reproductive organs treatment (PFT) using CCM.

Paper Title: Small temperature variations are a key regulator of reproductive growth and assimilate storage in oil palm (Elaeis guineensis)

Author list: Naoki Tani, Zubaidah Aimi Abdul Hamid, Natra Joseph, Othman Sulaiman, Rokiah Hashim, Takamitsu Arai, Akiko Satake, Toshiaki Kondo, Akihiko Kosugi

Supplemental Table 3. Estimated predictive skills (ρ) and standard deviation (SD) of ρ at every library size of causal relationship from meteorological to biological time-serieses on pruning female reproductive organs treatment (PFT) using CCM.

Time-series data		CCM result of the selected *tp* representing maximum ρ					CCM result of the selected *tp* same as NAT trees				
Library	Target	optimal *E*	selected *tp*	library size	ρ	SD of ρ	optimal *E*	selected *tp*	library size	ρ	SD of ρ
Starch in stem	CT (55 days)	8	4	8	0.385	0.157	8	7	8	0.328	0.181
		8	4	13	0.467	0.114	8	7	13	0.404	0.138
		8	4	18	0.503	0.073	8	7	18	0.456	0.107
		8	4	23	0.516	0.063	8	7	23	0.480	0.098
		8	4	28	0.525	0.053	8	7	28	0.472	0.098
		8	4	33	0.532	0.048	8	7	33	0.486	0.084
		8	4	38	0.537	0.040	8	7	38	0.488	0.071
Soluble sugar in stem	CT (59 days)	8	-6	8	0.338	0.143	8	-7	8	0.325	0.156
		8	-6	13	0.426	0.128	8	-7	13	0.407	0.147
		8	-6	18	0.485	0.108	8	-7	18	0.466	0.124
		8	-6	23	0.522	0.092	8	-7	23	0.514	0.096
		8	-6	28	0.559	0.077	8	-7	28	0.546	0.089
		8	-6	33	0.580	0.070	8	-7	33	0.578	0.072
		8	-6	38	0.591	0.077	8	-7	38	0.584	0.072
Starch in stem	CR (60 days)	3	8	3	0.069	0.160	3	-1	3	-0.004	0.093
		3	8	8	0.172	0.153	3	-1	8	0.040	0.109
		3	8	13	0.212	0.127	3	-1	13	0.064	0.103
		3	8	18	0.228	0.120	3	-1	18	0.066	0.098
		3	8	23	0.246	0.107	3	-1	23	0.084	0.100
		3	8	28	0.272	0.088	3	-1	28	0.109	0.097
		3	8	33	0.281	0.084	3	-1	33	0.101	0.096
		3	8	38	0.283	0.074	3	-1	38	0.121	0.085
Soluble sugar in stem	CR (60 days)	8	4	8	0.297	0.153	8	3	8	0.307	0.156
		8	4	13	0.390	0.140	8	3	13	0.414	0.147
		8	4	18	0.430	0.121	8	3	18	0.441	0.124
		8	4	23	0.440	0.121	8	3	23	0.443	0.096
		8	4	28	0.486	0.090	8	3	28	0.478	0.089
		8	4	33	0.496	0.095	8	3	33	0.485	0.072
		8	4	38	0.511	0.082	8	3	38	0.499	0.072
